# Long-term mental health and social support in victims of disaster: comparison with a general population sample

**DOI:** 10.1192/bjo.2018.74

**Published:** 2018-12-21

**Authors:** Siri Thoresen, Marianne Skogbrott Birkeland, Filip K. Arnberg, Tore Wentzel-Larsen, Ines Blix

**Affiliations:** Research Professor, Norwegian Centre for Violence and Traumatic Stress Studies, Norway; Senior Researcher, Norwegian Centre for Violence and Traumatic Stress Studies, Norway; Associate Professor, National Centre for Disaster Psychiatry, Department of Neuroscience, Psychiatry, Uppsala University, Sweden; Senior Researcher, Norwegian Centre for Violence and Traumatic Stress Studies, Norway and Centre for Child and Adolescent Mental Health, Norway; Senior Researcher, Norwegian Centre for Violence and Traumatic Stress Studies, Norway.

**Keywords:** Disaster, mental health, social functioning, long term

## Abstract

**Background:**

Trauma and traumatic bereavement have well-known consequences for mental health, but little is known about long-term adjustment, particularly with respect to health-protective factors.

**Aims:**

To assess the levels of anxiety/depression and perceived social support among the survivors and the bereaved 26 years after the Scandinavian Star ferry disaster compared with expected levels from the general population.

**Method:**

Anxiety/depression and social support were assessed in face-to-face interviews with the survivors and the bereaved (*N* = 165, response rate 58%). Expected scores were calculated for each participant based on the means and proportions for each age and gender combination from a general population sample. We computed the ratio between expected and observed scores, standardised mean differences with 95% confidence intervals and standardised effect sizes.

**Results:**

We found an elevated level of anxiety/depression symptoms in the victims (*M*_diff_ = 0.28, 95% CI 0.18, 0.38; effect size 0.43, 95% CI 0.31, 0.55) and a significant excess of individuals with a clinically significant level of symptoms. The observed level of perceived social support was significantly lower than that expected (*M*_diff_ = −0.57, 95% CI −0.70, −0.44; effect size −0.73, 95% CI −0.89, −0.57). This was the case for both survivors and those who were bereaved and for both men and women.

**Conclusions:**

This study reveals that disaster survivors and the bereaved reported elevated levels of anxiety and depression symptoms 26 years after the event. They also reported a markedly reduced level of social support. Traumas and post-traumatic responses may thus cause lasting harm to interpersonal relationships.

**Declaration of interest:**

None.

Disasters can have negative mental health consequences for the victims in the early and intermediate aftermath,^1^ with symptoms reaching their peak during the first year after the event.^2^ However, little is known about the long-term effects on health as few studies have conducted follow-up assessments more than 2 years after a disaster.^3^ In recent years, there has been a focus on early interventions and early responses to disasters. However, to provide effective care, it is also necessary to understand how people adapt to extreme stress over time.^4^ The few previous studies of long-term consequences of disasters suggest that victims suffer prolonged mental health consequences.^5^^,^^6^ For example, a threefold increase in the risk of mental health problems was found for survivors three decades after an oil platform collapse.^7^ That said, there are also some studies that have shown modest effects. For example, a 20-year follow-up of children exposed to an Australian bushfire found only a small impact on adult psychiatric morbidity^8^ and parents who lost their son in a military training accident seemed to be in good health at a 23-year follow-up, despite exhibiting high symptom levels early on.^9^ The lack of studies and their variable findings raise the question of whether long-term health problems among disaster victims exceed the levels found in the general population. Even less is known about how traumatic experiences or traumatic bereavement affect health-protective factors in the long term. Social relationships are known to have important implications for long-term health.^10^ Researchers often refer to three main areas of social relationships: social connectedness, social networks (such as the structure, size and frequency of contact) and received and perceived social support.^11^ In particular, perceived social support has been found to be an important buffer against negative health development after adversity,^12^^,^^13^ protecting against both post-traumatic stress and general mental and physical health problems.^10^ However, it is debated whether social support deteriorates over time among trauma survivors with prolonged symptoms.^14^^,^^15^

The purpose of this study was to compare the level of anxiety/depression symptoms and perceived social support among the survivors and the bereaved 26 years after a ferry disaster with expected levels derived from a general population sample.

## Method

### Samples and procedures

In 1990, a fire occurred on the Scandinavian Star passenger ferry, killing 159 of the 482 people on board (33%). Because the majority of the passengers were either families on vacation or athletic clubs on their way to training camps, many of the victims were young people. Although the police concluded that the fire was most likely arson, the perpetrator(s) was never identified.

In 2016, the Norwegian Parliament requested a systematic evaluation of the mental health of the survivors and the bereaved as part of a broader investigation into the cause and consequences of the event. The commission supplied a list of survivors from the ship and a list of the bereaved who received compensation settlements from the ship owners' insurance company.^16^ At the time of the study, 321 Norwegian survivors (*N* = 163) and bereaved (*N* = 158) were alive and traceable. They were sent postal information letters and those who did not opt out were contacted by phone. Face-to-face interviews were conducted between September and December 2016 by healthcare professionals who had attended a 1-day training seminar. Participants (*N* = 193) gave their written consent to the inclusion of their information in the report to the Norwegian government, and most participants (96%, *N* = 185) gave an additional written consent to use the information for research purposes. In total, 185 of the 321 individuals we attempted to reach participated in the study (94 survivors and 91 bereaved), yielding a response rate of 58% for both groups. Participants were classified as ‘survivors’ (present on the ship at the time of the fire) and ‘bereaved’ (not present on the ship but lost a close relative in the fire). Some survivors (32.2%) also lost someone they knew in the fire; however, only a small minority lost a close family member (6.9%). The traumatic exposure was severe for many of the survivors as 76% were in areas of the ship with heavy smoke, 42% heard people screaming or calling for help, 36% saw injured persons or bodies of deceased persons and 62% experienced a dangerous situation during the evacuation of the ship.^16^ Of the bereaved, 86% (*n* = 78) lost one, two or three close family members in the fire (i.e. partners, children, siblings, parents, grandchildren or grandparents). Of the remaining 13 individuals, 10 lost other relatives and 3 lost ex-partners or others.

The Regional Committee for Medical and Health Research Ethics approved the study (registration number 2016/1527). To ensure confidentiality, the participants' responses were recorded on a tablet and transferred via secure encryption to a dedicated server for sensitive data. The study also included a follow-up service for participants in distress. The research team cooperated with the national disaster support group in the design of the study and the training of the interviewers.

The general population sample was collected in 2013 to estimate the national prevalence of exposure to violence. To this end, a representative sample of the population aged between 18 and 74 years was drawn from the General Population Registry of Norway. Potential participants were sent postal invitation letters. Individuals were called randomly from the population registry sample, and the calling ended when the pre-specified sample size was achieved. In total, 13 794 did not answered the phone, leaving 9647 individuals who answered and were asked to participate in the study. Of these, 5120 declined to participate and 4527 agreed to participate. Not including unidentified telephone numbers and unanswered phone calls, which is comparable to the random digit dialling procedures, the response rate was 42.9% (45.0% female and 40.8% male). Further details regarding the participants and research methods are published elsewhere.^17^

#### Individuals eligible for comparison

The age range in the Scandinavian Star sample (*N* = 185) was 27–89 years, whereas the ages of those included in the general population sample ranged from 18 to 74 years. Thus, 19 participants from the disaster sample who were aged ≥75 years and 1 participant from the disaster sample who had an undisclosed age were excluded, resulting in 165 disaster-exposed individuals eligible for our analyses. The Scandinavian Star sample did not differ significantly from the comparison sample with respect to financial status or education level (χ^2^ *P*-values ranging from 0.213 to 1.000) and we accounted for age and gender in the analyses.

### Measures

Symptoms of anxiety and depression during the past week were measured using the ten-item version of the Hopkins Symptom Checklist (HSCL-10).^18^ The ten symptoms included feeling hopeless about the future; feeling sad; experiencing self-blame; feeling everything is an effort; feeling worthless; becoming suddenly scared for no reason; feeling faint, dizzy or weak; feeling fearful; feeling tense or anxious and having difficulties falling asleep or staying asleep. Responses were recorded on a scale from 1 (not bothered) to 4 (bothered a great deal). This screening measure exhibits good psychometric properties and is strongly correlated (*r* = 0.97) with the HSCL-25 in a general population sample.^19^ Cronbach's alpha was 0.93 for the disaster sample and 0.89 for the general population sample. A mean anxiety/depression score was calculated for each participant. For six participants with one missing item each, we calculated the mean of the nine other items. An HSCL-10 cut-off score of ≥1.85 was used to indicate a high level of anxiety/depression symptoms.^20^

Perceived social support was measured using the Crisis Support Scale,^21^ which included the following four questions: ‘when you feel the need to talk, how often is someone willing to listen to you?’, ‘are you able to talk about your thoughts and feelings?’, ‘do people show you sympathy and support?’ and ‘is there someone who can give you practical help?’ Responses were recorded on a scale from 1 (never) to 5 (very often/always). Cronbach's alpha was 0.79 for the disaster sample and 0.70 for the general population sample. A mean perceived social support score was calculated for participants with four (*n* = 160) and three (*n* = 4) valid items, and one participant with two missing items was excluded from the analyses.

### Statistical analyses

We calculated the expected anxiety/depression and perceived social support scores for each participant in the disaster sample (*N* = 165) based on the means for each age and gender combination from the general population sample. For example, a 57-year-old male participant from the disaster sample was ascribed an expected score based on the mean of the male 57-year-old participants in the general population sample. The general population sample included 3694 individuals within the disaster sample age range, and the number within each gender and age combination in the general population sample ranged from 12 to 67.

We computed the expected proportion above the cut-off for each participant in the disaster sample as the observed proportions ≥1.85 in each age and gender combination in the general population sample. We did not conduct any similar analyses for perceived social support as there was no agreed-upon cut-off value indicating low support.

We present the disaster sample participants' observed scores, expected scores, and the ratio between their observed and expected scores for anxiety/depression and perceived social support. Standardised effect sizes are reported as the standardised mean difference between the observed and the expected scores using the standard deviation of the observed scores.

For mean differences between observed and expected scores of anxiety/depression and perceived social support, between observed and expected proportions above cut-off for anxiety/depression and for standardised effect sizes, 95% confidence intervals were computed using the bootstrap-percentile procedure both for the total sample and separately for each gender, as well as separately for survivors and bereaved participants. The computations were based on 10 000 bootstrap resamples, drawn separately within each gender in the general population sample and within each combination of gender and survivors/bereaved participants within the participants from the disaster sample. Expected scores were recomputed within each bootstrap resample to take into account random variation within the general population sample as well.

The R software was used for all analyses (The R Foundation for Statistical Computing, Vienna, Austria, https://www.r-project.org/) with the packages boot (https://CRAN.R-project.org package=boot, maintainer Brian Ripley) for bootstrapping and psy (https://cran.r-project.org/web/packages/psy/index.html, maintainer Bruno Falissard) for computations of Cronbach's alpha.

## Results

The disaster sample (*N* = 165) included 51.5% (*n* = 85) women and 48.5% (*n* = 80) men with a mean age of 52.5 years (range 27–74 years). Approximately half of the participants were survivors (52.7%, *n* = 87), whereas the others were bereaved (47.3%, *n* = 78). The majority were married or living with a romantic partner (68.5%, *n* = 113), had 16 or more years of education (54.5%, *n* = 90) and perceived their financial status to be average or above average (88.3%, *n* = 144).

### Anxiety/depression

The observed score for anxiety/depression symptoms in the disaster sample was 1.58, the expected score was 1.31 and the mean ratio between the observed and expected scores was 1.21. The mean difference between the observed and expected scores was 0.28 (95% CI 0.18, 0.38). The estimated effect size of the difference was 0.43 (95% CI 0.31, 0.55). Although the bereaved reported a somewhat higher level of anxiety/depression symptoms (*M*_survivors_ = 1.47 [s.d. = 0.57], *M*_bereaved_ = 1.67 [s.d. = 0.69], *t*-test *P* = 0.031), perceived social support did not differ significantly between the groups (*P* = 0.624). There were no significant gender differences in anxiety/depression or perceived social support (*P* = 0.182–0.689).

The proportion in the disaster sample above the cut-off for anxiety/depression symptoms was 0.27 (95% CI 0.21, 0.34), the estimated expected proportion was 0.11 (95% CI 0.10, 0.12) and the difference was 0.16 (95% CI 0.10, 0.23). This indicates a significant excess of individuals with clinically significant anxiety/depression symptoms in the disaster sample.

**Fig. 1:**
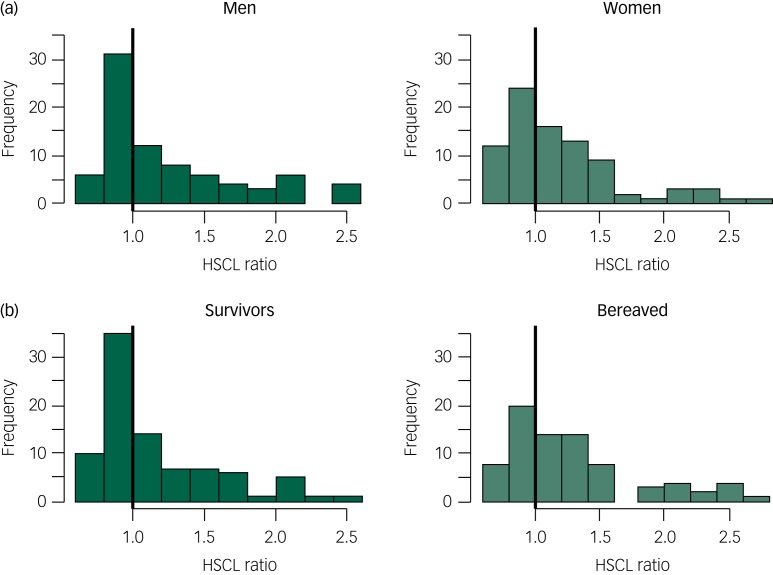
Ratio between observed and expected anxiety/depression scores (HSCL) in men and women (a) and in the survivors and the bereaved (b).

Figure 1a illustrates the ratios between observed and expected anxiety/depression scores for men and women separately. A ratio of 1 represents a symptom level equal to the expected score. Participants on the left side of the vertical black line reported lower-than-expected anxiety/depression scores and participants on the right side reported higher scores than those expected. The excess level of anxiety/depression symptoms among the disaster victims was significant for both men (*M*_diff_ = 0.30; 95% CI 0.16, 0.44) and women (*M*_diff_ = 0.26; 95% CI 0.13, 0.40). As illustrated in Fig. 1b, a higher-than-expected level of anxiety/depression symptoms was found for both the survivors (*M*_diff_ = 0.19; 95% CI 0.07, 0.31) and the bereaved (*M*_diff_ = 0.38; 95% CI 0.23, 0.53).

### Perceived social support

**Fig. 2:**
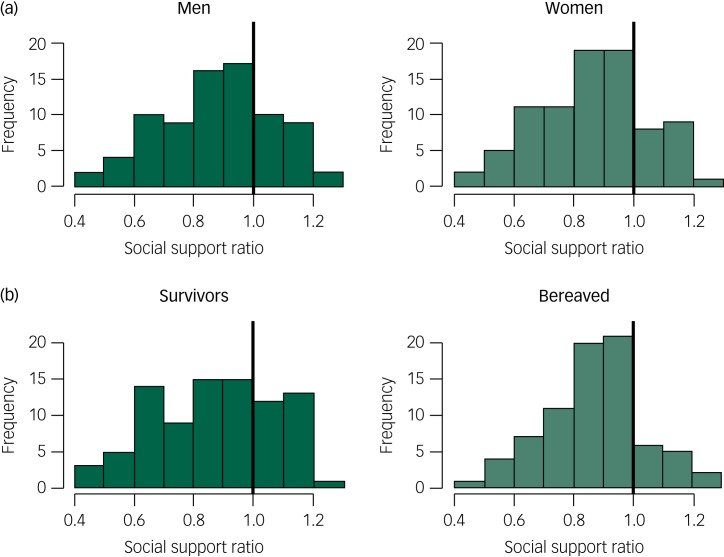
Ratio between observed and expected perceived social support in men and women (a) and in the survivors and the bereaved (b).

The observed mean score for perceived social support in the disaster sample was 3.72, the expected score was 4.29 and the mean ratio between the observed and expected scores was 0.87. The mean difference between the observed and expected scores was −0.57 (95% CI −0.70, −0.44), which corresponded to an effect size of −0.73 (95% CI −0.89, −0.57). A lower-than-expected level of perceived social support was observed for both men (*M*_diff_ = −0.53; 95% CI −0.71, −0.35) and women (*M*_diff_ = −0.61; 95% CI −0.78, −0.44) (Fig. 2a). Both the survivors (*M*_diff_ = −0.57; 95% CI −0.75, −0.39) and the bereaved (*M*_diff_ = −0.58; 95% CI −0.74, −0.41) reported lower-than-expected levels of perceived social support (Fig. 2b).

## Discussion

The survivors and the bereaved reported elevated levels of anxiety and depression symptoms and decreased levels of perceived social support; this was true for both men and women. Our study is the first to investigate, from a long-term perspective, both mental health and perceived social support in people who survived and were bereaved by a traumatic event. Our results contribute to the scarce literature on the long-term consequences of disasters.

### Anxiety/depression

The difference in anxiety/depression symptoms between the victims' observed and expected scores was of a moderate effect size,^22^ despite the many years that had passed since the traumatic event. Previous studies of the Danish Scandinavian Star survivors revealed a high level of symptoms and a lack of recovery at an early stage.^23^^,^^24^ Although some disaster victims reported a lower-than-expected symptom level, there was a substantial excess of victims reporting clinically significant anxiety/depression symptoms. The 27% caseness observed in this study is strikingly similar to the few other long-term studies of comparable disasters. Fifteen years after the Estonia ferry disaster in the Baltic Sea, 27 years after an oil rig disaster in the North Sea and 14 years after the Buffalo Creek dam collapse, 20–28% of the victims suffered from severe mental health problems.^7^^,^^25^^,^^26^ Nevertheless, other studies indicate no or only modest increases in mental health problems over the long term.^8^^,^^9^ The reasons for these discrepant results are uncertain and it is unknown what effects the type of disaster (such as man-made versus natural disasters) or characteristics of the event (such as property destruction or loss of life) have on the long-term mental health burden.^1^ The difference between man-made and natural disasters is not always clear as natural forces may play an important role in man-made disasters, and man-made infrastructure, housing and safety protection (or lack thereof) may be decisive with respect to the impact of a natural disaster. What the above-mentioned disasters all have in common is that they resulted in a high number of casualties. Further research is necessary to determine which aspects of a disaster are key to the future burden of those affected.

The results of these long-term studies are compatible with the resilience literature which emphasises that the majority of trauma-exposed individuals do not develop prolonged mental health problems.^27^ Nevertheless, it is important to keep in mind that a considerable minority of survivors and bereaved will carry a lifetime burden of anxiety, depression or post-traumatic stress. Because of the frequency and scope of disasters worldwide, a substantial number of people are at risk of very prolonged suffering.

### Perceived social support

We found a markedly reduced level of perceived social support among the survivors and bereaved alike, with a moderate to large effect size. Perceived social support has a well-documented link to both mental and physical health^10^ and is considered to be the most important protective factor following adversity.^12^^,^^13^ Consistent with the buffer hypothesis, an initially high level of perceived social support predicts a lower level of mental health problems after a trauma^28^^–^^30^ as well as a more rapid recovery among those who develop symptoms.^31^^,^^32^ However, recent research has led to changes in how we perceive the links between adversity, social support and mental health, and some studies indicate that social support may deteriorate over time following a traumatic event.^14^^,^^15^^,^^33^ Post-disaster displacement and community disruption have been proposed as explanations for such social support deterioration.^1^ In the present study, the disaster victims returned to an intact home and community, indicating that more psychological or relational processes may be at work.

The processes leading to potential disturbances in the social relationships of disaster victims and their links to mental health are likely complex and not well understood. Mental health problems may interfere with social skills^34^ and post-traumatic loss, bitterness or frustration may result in a negative shift in network orientation.^35^ Unsupportive responses from others, such as blaming or being let down, are not uncommon among trauma victims^36^ and may further increase negative social expectations. As a result, the victims may stop seeking social support or their social support providers may withdraw from them. These processes may harm the social relationships, causing the victim to reduce his or her engagement with the social network in terms of frequency or variety of contacts, and potentially lead to a lack of social connectedness, which again exacerbates their mental health problems.^37^

In the aftermath of traumatic events, the initial mobilisation of social support may be withdrawn long before the victims have recovered,^6^ causing the survivors who fail to recover during the first year to experience social rejection.^14^ In a previous publication, we demonstrated that a substantial number of survivors and bereaved in the current sample, even after 26 years, entertained vivid thoughts about the disaster and what could have happened, which may be at odds with the expectations of significant others.^38^ Additionally, the victims may refrain from taking advantage of the social support available, sometimes because they fear that others will perceive them as weak or because they think they will overburden their friends and families.^6^^,^^39^^,^^40^ In addition, family and friends may feel uncomfortable or find it difficult to relate to the victims, withdrawing from them as a result.^41^ This study is the first to indicate that social support deterioration may last for decades and proposes that even though social support can be considered a protective factor in the initial phase after a trauma, social support is also a long-term outcome in itself.

The excess level of anxiety/depression symptoms and the reduced level of perceived social support were significant for both men and women, and for both the bereaved and the survivors. This does not necessarily mean that these groups suffer in the same manner, have the same symptoms or have the same relational difficulties. As the use of mean scores may mask important differences between the groups, further research is necessary to identify the unique long-term development in men and women, as well as in the bereaved and survivors, in the aftermath of disasters.

### Strengths and limitations

This cross-sectional study could not identify causal links or determine how perceived social support and anxiety/depression have evolved over time. Furthermore, we could not compare levels of post-traumatic stress symptoms because a general population sample will include individuals without a history of traumatic exposure. Although we did not identify socioeconomic differences between the disaster sample and the comparison sample, the comparison sample may nonetheless have been biased and the estimated differences should thus be interpreted with caution. Previous analyses have indicated a small selection bias in the general population sample in terms of slightly higher income and education.^17^ However, we could not identify any significant differences between the general population sample and the disaster sample in education or income, and our analyses adjusted for age and gender. The disaster sample may have been biased in several ways, for example, with respect to health status and survival in the 26 years that had elapsed. Non-response may also have been associated with health as healthy individuals may have considered the study irrelevant for them, whereas individuals with excessive symptoms may have found participation too distressing. Previous research has investigated whether disaster-related health problems are related to study participation, but the results are inconclusive.^42^^,^^43^ Our previous investigation indicates that the current sample seemed to be fairly representative in relation to exposure level (survivors) and relations to the deceased (bereaved).^16^

The HSCL is a screening measure with some uncertainty attached to the cut-off value. Although the Crisis Support Scale intends to measure the emotional, cognitive and instrumental aspects of perceived social support, the single items are somewhat heterogeneous and the internal consistency was somewhat low in the general population sample (0.70). The measure may tap in to both the ability to engage with other people and the availability and responsiveness of other people. Therefore, the reduced level of perceived social support among the disaster victims may reflect negative changes in an individual's network orientation, social skills or impairments occurring in the individual's social network, or a combination of negative changes in the individual and in the social network. Other important aspects of social relationships such as received support, negative social support, social integration and social connectedness^11^ were not measured in this study.

We cannot rule out the possibility that different assessment methods have had some impact on the results. Both samples were primed on negative events, although the events differed (disaster versus violence). Furthermore, recalling the disaster may have influenced the respondents' mental states and thereby their responses to current mental health problems. All disasters occur in a specific context and this ferry disaster remains an unsolved crime whose aftermath is riddled with controversy. That said, unresolved issues and conflicts that may drag on for years are not uncommon in the aftermath of disasters.^44^ In addition, although some early crisis support was made available, there was no organised effort to provide healthcare to the victims. It is hoped that victims of more recent events have received more systematic interventions, although the health-promoting effects of such interventions are unknown. Several factors that were not addressed in this study may have affected the comparisons with the general population sample.

The strengths of this study included the high response rate of 58% even 26 years after the disaster, the face-to-face interview setting and the unique sample in which all the participants had been directly affected by the fire, either as survivors or as bereaved. Another strength was the availability of a comparison sample that allowed us to calculate expected scores. The considerable length of time that had passed since the disaster can be considered both a strength and a limitation.

In contrast to several other types of collective disasters such as hurricanes, floods and tsunamis, this disaster did not entail loss of physical resources (e.g. destroyed housing or infrastructure). Rather, the victims may have experienced other losses, including the loss of someone close and the loss of their sense of safety. These experiences may be compared to individual traumatic events that do not involve many other people. Therefore, our findings may be relevant not only in the context of a disaster or mass trauma but also for those experiencing individual trauma.

Our results underscore the long-lasting health and social consequences of disasters. Further research is needed to fully understand how trauma and post-trauma responses may interfere with social relationships, and future studies should include an investigation of social support or problems with social interactions as potential outcomes of trauma exposure. Particularly, it would be of value to disentangle the relationship between post-trauma responses, social support, social networks, connectedness and health. Clinicians may find it helpful to map trauma victims' current social support and focus on their social cognitions and social skills. Interventions that aim to ease interpersonal tension and resolve barriers to social support may also be beneficial to victims.

## References

[1] Goldmann E, Galea S. Mental health consequences of disasters. Annu Rev Public Health 2014; 35: 169–83. 24159920 10.1146/annurev-publhealth-032013-182435

[2] Norris FH, Friedman MJ, Watson PJ. 60,000 disaster victims speak: part II. Summary and implications of the disaster mental health research. Psychiatry 2002; 65: 240–60. 12405080 10.1521/psyc.65.3.240.20169

[3] Norris FH. Disaster research methods: past progress and future directions. J Trauma Stress 2006; 19: 173–84. 16612819 10.1002/jts.20109

[4] Galea S. The long-term health consequences of disasters and mass traumas. CMAJ 2007; 176: 1293–4. 17452663 10.1503/cmaj.070368 PMC1852853

[5] Hull AM, Alexander DA, Klein S. Survivors of the Piper Alpha oil platform disaster: long-term follow-up study. Br J Psychiatry 2002; 181: 433–8. 12411271 10.1192/bjp.181.5.433

[6] Arnberg FK, Hultman CM, Michel P-O, Lundin T. Fifteen years after a ferry disaster: clinical interviews and survivors’ self-assessment of their experience. Eur J Psychotraumatol 2013; 4: 10.3402/ejpt.v4i0.20650. 10.3402/ejpt.v4i0.20650 PMC3790912 24106579

[7] Boe HJ, Holgersen KH, Holen A. Mental health outcomes and predictors of chronic disorders after the North Sea oil rig disaster: 27-year longitudinal follow-up study. J Nerv Ment Dis 2011; 199: 49–54. 21206247 10.1097/NMD.0b013e31820446a8

[8] McFarlane AC, Van Hooff M. Impact of childhood exposure to a natural disaster on adult mental health: 20-year longitudinal follow-up study. Br J Psychiatry 2009; 195: 142–8. 19648546 10.1192/bjp.bp.108.054270

[9] Kristensen P, Heir T, Herlofsen PH, Langsrud Ø, Weisæth L. Parental mental health after the accidental death of a son during military service: 23-year follow-up study. J Nerv Ment Dis 2012; 200: 63–8. 22210364 10.1097/NMD.0b013e31823e5796

[10] Thoits PA. Mechanisms linking social ties and support to physical and mental health. JHSB 2011; 52: 145–61. 10.1177/0022146510395592 21673143

[11] Santini ZI, Koyanagi A, Tyrovolas S, Mason C, Haro JM. The association between social relationships and depression: a systematic review. J Affect Disord 2015; 175: 53–65. 25594512 10.1016/j.jad.2014.12.049

[12] Ozer EJ, Best SR, Lipsey TL, Weiss DS. Predictors of posttraumatic stress disorder and symptoms in adults: a meta-analysis. Psychol Bull 2003; 129: 52–73. 12555794 10.1037/0033-2909.129.1.52

[13] Brewin CR, Andrews B, Valentine JD. Meta-analysis of risk factors for posttraumatic stress disorder in trauma-exposed adults. J Consult Clin Psychol 2000; 68: 748. 11068961 10.1037//0022-006x.68.5.748

[14] Kaniasty K, Norris FH. Longitudinal linkages between perceived social support and posttraumatic stress symptoms: sequential roles of social causation and social selection. J Trauma Stress 2008; 21: 274–81. 18553415 10.1002/jts.20334

[15] Shallcross SL, Arbisi PA, Polusny MA, Kramer MD, Erbes CR. Social causation versus social erosion: comparisons of causal models for relations between support and PTSD symptoms. J Trauma Stress 2016; 29: 167–75. 27077494 10.1002/jts.22086

[16] Thoresen S, Birkeland MS, Wentzel-Larsen T, Blix I. Loss of trust may never heal. Institutional trust in disaster victims in a long-term perspective: associations with social support and mental health. Front Psychol 2018; 9: 1204. 30061852 10.3389/fpsyg.2018.01204 PMC6055587

[17] Thoresen S, Myhre M, Wentzel-Larsen T, Aakvaag HF, Hjemdal OK. Violence against children, later victimisation, and mental health: a cross-sectional study of the general Norwegian population. Eur J Psychotraumatol 2015; 6: 10.3402/ejpt.v6.26259. PMC4296052 25591729

[18] Derogatis LR, Lipman RS, Rickels K, Uhlenhuth EH, Covi L. The Hopkins Symptom Checklist (HSCL): a self-report symptom inventory. Behav Sci 1974; 19: 1–15. 4808738 10.1002/bs.3830190102

[19] Tambs K, Moum T. How well can a few questionnaire items indicate anxiety and depression? Acta Psychiatr Scand 1993; 87: 364–7. 8517178 10.1111/j.1600-0447.1993.tb03388.x

[20] Strand BH, Dalgard OS, Tambs K, Rognerud M. Measuring the mental health status of the Norwegian population: a comparison of the instruments SCL-25, SCL-10, SCL-5 and MHI-5 (SF-36). Nord J psychiatry 2003; 57: 113–8. 12745773 10.1080/08039480310000932

[21] Joseph S, Williams R, Yule W. Crisis support, attributional style, coping style, and post-traumatic symptoms. Pers Individ Diff 1992; 13: 1249–51.

[22] Cohen J. Statistical power analysis. Curr Dir Psychol Sci 1992; 1: 98–101.

[23] Elklit A, Andersen LB. SCANDINAVIAN STAR. En undersøgelse af de fysiske, psykologiske og sociale eftervirkninger af en katastrofe (Danish) [Scandinavian Star. An Investigation into the Somatic, Psychological, and Social Consequences of a Disaster]. Psykologisk Institut, Aarhus Universitet, 1994.

[24] Elklit A, Andersen LB, Arctander T. SCANDINAVIAN STAR. Anden del. De fysiske, psykologiske og sociale eftervirkninger 3½ år efter katastrofen (Danish). [Scandinavian Star. Second part. The somatic, psychological, and social consequences 3½ years after the disaster]. Psykologisk Institut, Aarhus Universitet, 1995.

[25] Arnberg FK, Eriksson NG, Hultman CM, Lundin T. Traumatic bereavement, acute dissociation, and posttraumatic stress: 14 years after the MS Estonia disaster. J Trauma Stress 2011; 24: 183–90. 21442665 10.1002/jts.20629

[26] Green BL, Lindy JD, Grace MC, Gleser GC, Leonard AC, Korol M, Buffalo Creek survivors in the second decade: stability of stress symptoms. Am J Orthopsychiatry 1990; 60: 43. 2305844 10.1037/h0079168

[27] Bonanno GA. Loss, trauma, and human resilience: have we underestimated the human capacity to thrive after extremely aversive events? Am Psychol 2004; 59: 20. 14736317 10.1037/0003-066X.59.1.20

[28] Arnberg FK, Hultman CM, Michel PO, Lundin T. Social support moderates posttraumatic stress and general distress after disaster. J Trauma Stress 2012; 25: 721–7. 23184348 10.1002/jts.21758

[29] Birkeland MS, Hansen MB, Blix I, Solberg Ø, Heir T. For whom does time heal wounds? Individual differences in stability and change in posttraumatic stress after the 2011 Oslo bombing. J Trauma Stress 2017; 30: 19–26. 28103399 10.1002/jts.22158

[30] Birkeland MS, Knatten CK, Hansen MB, Hem C, Heir T. Long-term relationships between perceived social support and posttraumatic stress after the 2011 Oslo bombing: a three-year longitudinal study. J Affect Disord 2016; 202: 230–5. 27267295 10.1016/j.jad.2016.05.037

[31] Charuvastra A, Cloitre M. Social bonds and posttraumatic stress disorder. Annu Rev Psychol 2008; 59: 301. 17883334 10.1146/annurev.psych.58.110405.085650 PMC2722782

[32] Birkeland MS, Nielsen MB, Hansen MB, Knardahl S, Heir T. Like a bridge over troubled water? A longitudinal study of general social support, colleague support, and leader support as recovery factors after a traumatic event. Eur J Psychotraumatol 2017; 8: 1302692. 28451070 10.1080/20008198.2017.1302692 PMC5399997

[33] Nickerson A, Creamer M, Forbes D, McFarlane A, O'Donnell M, Silove D, The longitudinal relationship between post-traumatic stress disorder and perceived social support in survivors of traumatic injury. Psychol Med 2017; 47: 115–26. 27670088 10.1017/S0033291716002361

[34] Kessler RC, Price RH, Wortman CB. Social factors in psychopathology: stress, social support, and coping processes. Annu Rev Psychol 1985; 36: 531–72. 3883893 10.1146/annurev.ps.36.020185.002531

[35] Clapp JD, Beck JG. Understanding the relationship between PTSD and social support: the role of negative network orientation. Behav Res Ther 2009; 47: 237–44. 19162260 10.1016/j.brat.2008.12.006 PMC2656396

[36] Davis RC, Brickman E, Baker T. Supportive and unsupportive responses of others to rape victims: effects on concurrent victim adjustment. Am J Community Psychol 1991; 19: 443–51. 1892138 10.1007/BF00938035

[37] Shevlin M, McElroy E, Murphy J. Loneliness mediates the relationship between childhood trauma and adult psychopathology: evidence from the adult psychiatric morbidity survey. Soc Psychiatr Psychiatr Epidemiol 2015; 50: 591–601. 10.1007/s00127-014-0951-8 25208908

[38] Blix I, Kanten AB, Skogbrott Birkeland M, Thoresen S. Imagining what could have happened: types and vividness of counterfactual thoughts and the relationship with posttraumatic stress reactions. Front Psychol 2018; 9: 515. 29731729 10.3389/fpsyg.2018.00515 PMC5920021

[39] Thoresen S, Jensen TK, Wentzel-Larsen T, Dyb G. Social support barriers and mental health in terrorist attack survivors. J Affect Disord 2014; 156: 187–93. 24398044 10.1016/j.jad.2013.12.014

[40] Smith AJ, Felix ED, Benight CC, Jones RT. Protective factors, coping appraisals, and social barriers predict mental health following community violence: a prospective test of social cognitive theory. J Trauma Stress 2017; 30: 245–53. 28644538 10.1002/jts.22197

[41] Dyregrov K. Micro-sociological analysis of social support following traumatic bereavement: unhelpful and avoidant responses from the community. Omega 2004; 48: 23–44.

[42] Hussain A, Weisaeth L, Heir T. Nonresponse to a population-based postdisaster postal questionnaire study. J Trauma Stress 2009; 22: 324–8. 19644976 10.1002/jts.20431

[43] Stene LE, Dyb G. Research participation after terrorism. An open cohort study of survivors and parents after the 2011 Utøya attack in Norway. BMC Res Notes 2016; 9: 57. 26830191 10.1186/s13104-016-1873-1 PMC4736239

[44] Bos CK, Ullberg S, Hart P. The long shadow of disaster: memory and politics in Holland and Sweden. Int J Mass Emerg Disasters 2005; 23: 5–26.

